# A System-Level Investigation into the Mechanisms of Chinese Traditional Medicine: Compound Danshen Formula for Cardiovascular Disease Treatment

**DOI:** 10.1371/journal.pone.0043918

**Published:** 2012-09-04

**Authors:** Xiuxiu Li, Xue Xu, Jinan Wang, Hua Yu, Xia Wang, Hongjun Yang, Haiyu Xu, Shihuan Tang, Yan Li, Ling Yang, Luqi Huang, Yonghua Wang, Shengli Yang

**Affiliations:** 1 Bioinformatics Center, College of Life Sciences, Northwest A&F University, Yangling, Shaanxi, China; 2 Institute of Chinese Materia Medica, China Academy of Chinese Medical Sciences, Beijing, China; 3 School of Chemical Engineering, Dalian University of Technology, Dalian, Liaoning, China; 4 Lab of Pharmaceutical Resource Discovery, Dalian Institute of Chemical Physics, Chinese Academy of Sciences, Dalian, Liaoning, China; Uni. of South Florida, United States of America

## Abstract

Compound Danshen Formula (CDF) is a widely used Traditional Chinese Medicine (TCM) which has been extensively applied in clinical treatment of cardiovascular diseases (CVDs). However, the underlying mechanism of clinical administrating CDF on CVDs is not clear. In this study, the pharmacological effect of CDF on CVDs was analyzed at a systemic point of view. A systems-pharmacological model based on chemical, chemogenomics and pharmacological data is developed via network reconstruction approach. By using this model, we performed a high-throughput *in silico* screen and obtained a group of compounds from CDF which possess desirable pharmacodynamical and pharmacological characteristics. These compounds and the corresponding protein targets are further used to search against biological databases, such as the compound-target associations, compound-pathway connections and disease-target interactions for reconstructing the biologically meaningful networks for a TCM formula. This study not only made a contribution to a better understanding of the mechanisms of CDF, but also proposed a strategy to develop novel TCM candidates at a network pharmacology level.

## Introduction

Cardiovascular diseases (CVDs) are the leading cause of death in the world. In 2008, about 17.3 million people died from CVDs, representing 30% of total global deaths. The number has been estimated to increase to 23.6 million by 2030 [1]. Although diverse drugs and medications have already been employed on CVDs, developing new therapeutic tools are still in urgent need and under intensive investigation. As one of these efforts, modernization of Traditional Chinese Medicine (TCM) has attracted a lot of attention [2].

Compound Danshen Formula (CDF) is one of TCM recipes for treatment of CVDs which is composed of *Radix Salviae Miltiorrhizae* (Labiatae sp. plant, Chinese name Danshen), *Panax Notoginseng* (Araliaceae plant, Chinese name Sanqi), and *Borneolum* (Crystallization of the resin and volatile oil in Cinnamomum camphora (L.) Presl, Chinese name Bingpian), at a ratio of 450∶141∶8 (g) [3]. CDF is officially registered in Chinese Pharmacopoeia [3] and has been widely used to treat CVDs in China, Japan, United States and Europe [4]. Clinical studies have revealed a variety of desirable pharmacological effects of CDF on CVD, such as increasing coronary flow rate, activating superoxide dismutase, dilating coronary vessels etc, which contribute significantly to the survival rate of CVD patients [5]–[7]. However, the molecular details about how CDF can be administrated on CVD are still unclear.

Studies on CDF's pharmacological effect have confronted several major challenges. First, isolation and identify chemical constituents possessing desirable pharmacological effects are labor-intensive, time-consuming and costly, given the fact that most medicinal herbs may contain tens of thousands constituents. Second, a certain ingredient may function on several relevant or irrelevant biological targets, which makes its pharmacological and toxicological effects difficult to be evaluated independently. Third, and most importantly, TCMs, such as CDF, have traditionally been administrated as an integrated prescription for treating diseases which implicate a complex, and highly dynamic ingredient-ingredient interaction network may underlying the overall clinical effect [8], [9].

Systems pharmacology has emerged as a promising subject to overcome these challenges by providing powerful new tools and conceptions. Network analysis is one of these approaches which can evaluate TCM's pharmacological effect as a whole unity [10]–[14]. In this work, we proposed for the first time a systems-pharmacological model by combining oral bioavailability prediction, multiple drug-target prediction and validation, and network pharmacology techniques, to shed new lights on the effectiveness and mechanism of CDF. Different types of data, such as the physiological, biochemical and genomic information have been collected to build the model which is based on an array of computational approaches including the machine learning method and network analysis. The proposed network-driven, integrated approach would also provide a novel and efficient way to deeply explore the chemical and pharmacological basis of TCMs.

## Materials and Methods

As a combination of three plants, CDF contains a considerable number of chemical compounds and some of which have been demonstrated to possess significant pharmacological activities [15]–[20]. This provides an important basis to bring systems biology insights into the investigation of TCM theory and practice. In the following part, we will introduce how to build database and models for this CDF.

### Database construction

**Figure 1:**
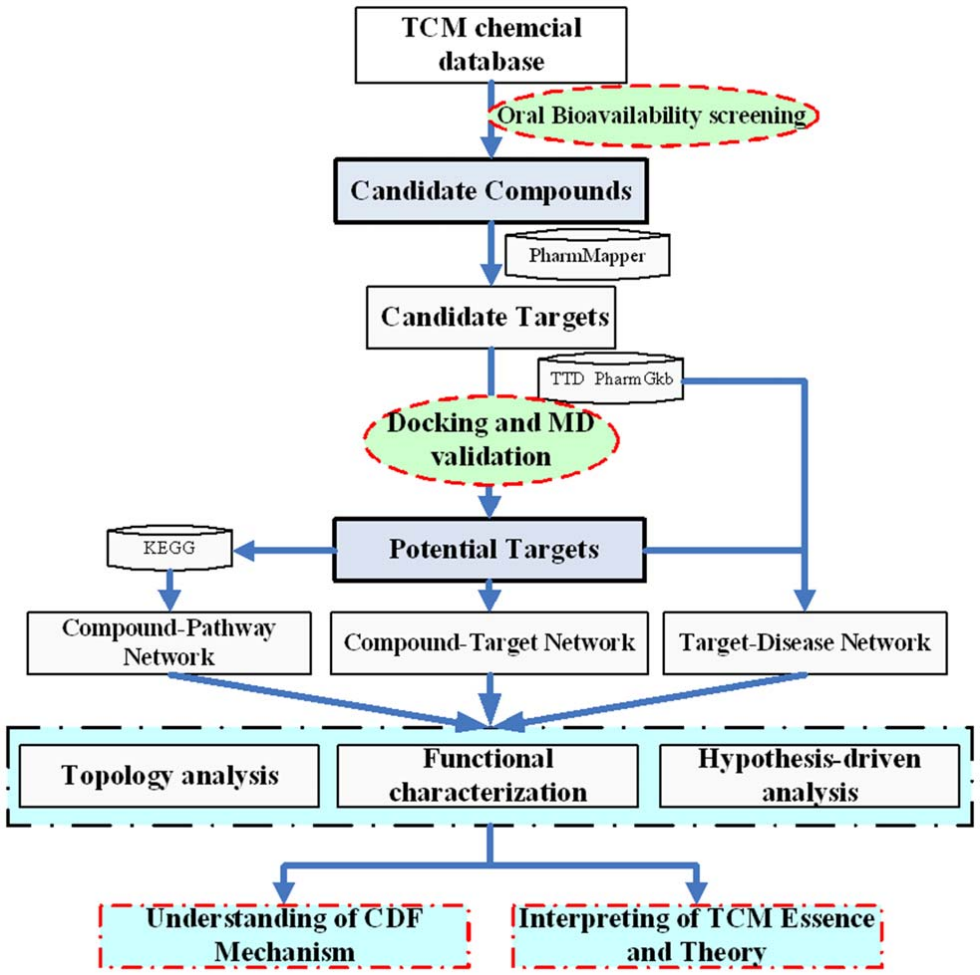
Flowchart of the model building.

All chemicals of each herb were retrieved from Chinese Academy of sciences Chemistry Database (www.organchem.csdb.cn) and Chinese Herbal Drug Database [21] and literature [15]–[17]. Finally, to the most extent 320 compounds were collected, including 201 in *Radix Salviae Miltiorrhizae*, 112 in *Panax Notoginseng* and 31 in *Borneolum*, respectively (The three herbal shared the same 24 compounds). The structures of these molecules were downloaded from LookChem (www.lookchem.com) or produced by ISIS Draw 2.5 (MDL Information Systems, Inc.) and further optimized by Sybyl 6.9 (Tripos, Inc) with sybyl force field and default parameters [22], [23]. The molecules were saved to a mol format for further analysis. All information about the molecules is provided in Table S1. Figure 1 depicts the flowchart of the modeling procedure.

### Oral bioavailability prediction

In our previous work, we have developed a robust *in silico* model OBioavail 1.1 [24], which integrated with the metabolism information to predict a compound human oral bioavailability. The model was built based on a set of 805 structurally diverse drug and drug-like molecules which have been critically evaluated for their human oral bioavailability [25]. The multiple linear regression, partial least square and support vector machine (SVM) methods were employed to build the models, resulting in an optimal model with R^2^ = 0.80, SEE = 0.31 for the training set, Q^2^ = 0.72, SEP = 0.22 for the independent test set. In this work, the compounds with OB ≥50% were selected as the Candidate Compounds (Figure 1). The threshold determination is based upon the careful consideration of the following rules: 1) Extracting information as much as possible from CDF using the least number of compounds. 2) The obtained model can be reasonably interpreted by the reported pharmacological data.

### Target identification

The targets were searched by PharmMapper Server (http://59.78.96.61/pharmmapper/) [26], which is designed to identify potential target candidates for the given small molecules (drugs, natural products, or other newly discovered compounds with targets unidentified) via a ‘reverse’ pharmacophore mapping approach. The model is supported by a large repertoire of pharmacophore database composed of more than 7,000 receptor-based pharmacophore models that are extracted from TargetBank, DrugBank, BindingDB and PDTD. A strategy algorithm of sequential combination of triangle hashing and genetic algorithm optimization is designed to solve the molecule pharmacophore best fitting task. In this work, the number of the reserved matched targets is defined as 300 with the fitting score ≥3.00. The target set is only limited to the human targets (2214); and all parameters were kept as default. The information of the predicted target candidates which have relationships with CVD was collected and further verified from TTD (http://bidd.nus.edu.sg/group/ttd/) [27], PharmGkb (www.pharmgkb.org) [28] and DrugBank (http://www.drugbank.ca/) [29].

### Target validation

#### Docking

To validate the compound-target associations related with CVD, the molecular docking simulation was further performed on each bioactive compound complexed with their human target enzymes by AutoDock software (version 4.2, http://autodock.scripps.edu/) (Figure 1). All the protein structures except P-glycoprotein (P-gp) were directly downloaded from the RCSB protein data bank (www.pdb.org) [30] with their resolutions being carefully checked. The homology model of P-gp was obtained from our previous work [31]. AutoDock tools (ADT) (version 1.4.5) were used for protein and ligand preparation. Generally, all hydrogens, including non-polar, Kollman charges and solvation parameters were added to individual molecules. For all ligands, the Gasteiger charges [32] were assigned with the nonpolar hydrogens merged [33]. The auxiliary program Autogrid was used to generate the grid maps for each sample. The docking area was defined by a 60×60×60 3D grid centered around the ligands binding site with a 0.375 Å grid space. All bond rotations for the ligands were ignored and the Lamarckian genetic algorithm (LGA) was employed for each simulation process.

#### Molecular dynamics simulation

All molecular dynamics simulations were carried out using the Amber 10 suite of programs [34]. The standard AMBER99SB force field was selected for proteins [35], the ligand charges and parameters were determined with the antechamber module of Amber based on the AM1-BCC charge scheme [36] and the general atom force field (GAFF) [37]. All models were solvated in the rectangular box of TIP3P water extending at least 10 Å in each direction from the solute, and neutralized by adding sufficient Na^+^/Cl^−^ counterions. The cut-off distance was kept to 8 Å to compute the nonbonded interactions. All simulations were performed under periodic boundary conditions, and the long-range electrostatics were treated by using the Particle-mesh-Ewald method (PME) [38]. All bonds containing hydrogen atoms were fixed using the SHAKE algorithm.

After initial configuration construction, a standard equilibration protocol was performed for MD simulations. The systems were minimized by 500 steps of steepest descent and 1000 steps of conjugate gradient to remove the bad contacts in the structure, then were slowly heated to 300 K over 50 ps using 2.0 kcal/mol/Å^−2^ harmonic restraints. Subsequently, a 50 ps pressure-constant (1 bar) period to raise the density while still keeping the complex atoms constrained and a 500 ps equilibration were conducted. The production stage consisted of a total of 5 ns at constant temperature of 300 K for each system, respectively. The integration time step was 2 fs and the coordinates were saved every 2 ps.

#### Binding free energy calculation

The energy of the protein–ligand binding was computed using the Molecular Mechanics-Poisson Boltzmann Surface Area (MM-PBSA) methodology [39] by the MM-PBSA, SANDER and NMODE modules in Amber. In this approach, the frame of a MD trajectory was stripped off counterions and water molecules, and the binding free energy (ΔG_bind_) was calculated according to the following equations:
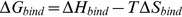
(1)

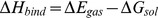
(2)

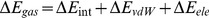
(3)

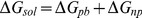
(4)

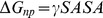
(5)where ΔE_gas_ is the molecular mechanical gas-phase energy, which is the sum of the internal (ΔE_int_), van der Waals (ΔE_vdw_) and electrostatics (ΔE_ele_) energies. The solvation free energy (ΔG_sol_) was calculated with a PB/SA model, which dissects the solvation energy in two parts, that is, the electrostatic (ΔG_pb_) and the nonpolar (ΔGnp) components. The ΔG_pb_ was calculated using the PBSA program with the default cavity radii. The dielectric constant was set to 2 for the interior solute and 80 for the surrounding solvent. The ΔG_np_ was computed based on eq. (5), where γ represents surface tension and was set to 0.0072 kcal·mol^−1^Å^−2^, and SASA is the solvent-accessible surface area (Å^2^) determined using the linear combination of pairwise overlaps model [40]. The entropy contributions (TΔS_bind_) arising from changes in the translational, rotational and vibrational degrees of freedom were calculated using statistical mechanics formulae [41]. Because the contributions from translation and rotation are much smaller than vibration, TΔS_bind_ was generally calculated using normal-mode analysis by the NMODE module in Amber.

### Network construction

The Candidate Targets and Potential Targets were respectively used to build the Compound-Target Networks with the Candidate Compounds. The Compound-Target Networks were generated by Cytoscape 2.8.1 [42], a standard tool for integrated analysis and visualization of biological networks. The Compound-Pathway Network was produced by linking the Candidate Compounds and the signal pathways in which they participated. The diseases related with the Potential Targets were collected from the PharmGkb, TTD and DrugBank databases and the obtained disease-target interactions were further applied to build the Target-Disease Network.

In the graphical networks, nodes represent the compounds, proteins, signal pathways or diseases, and edges encode the compound-target, compound-pathway or target-disease interactions. The “Compound-candidate Target Network” (C-cT Network) was constructed by linking the Candidate Compounds and all their Candidate Targets, while the “Compound-Potential Target Network” (C-T Network) was built by linking the Candidate Compounds and their validated Potential Targets. In the “Compound-Pathway Network” (C-P Network), the signal pathway was linked to a Candidate Compound if the compound target exists in the pathway. In the “Target-Disease Network” (T-D Network), the diseases were connected with those related Candidate Targets. Finally, the quantitative properties of these networks were analyzed by two plugins including NetworkAnalyzer [43] and CentiScaPe 1.2 [44].

## Results and Discussion

TCM prescriptions usually contain several herbs called “Fufang” in Chinese based on the principle of “Jun-Chen-Zuo-Shi” [45]. Up to today, more than 300 complex formulations prescribed in accordance to this theory have been in use for centuries [46]. And much effort has been made in proving the TCM efficacy by the criteria of evidence-based medicine or experience-based medicine [47]–[50], but the complexity of the chemical components would make it extremely difficult to understand this TCM principle from a molecular or systematic level. Therefore, in addition to the specific focus on the issue of CDF, the present work also attempts to interpret this formulation theory of TCM through this relatively simple recipe.

### Oral bioavailability prediction

Oral bioavailability, one of the most important pharmacokinetic parameters among ADME properties (absorption, distribution, metabolism and excretion), represents the percentage of an oral dose that is enough to produce a pharmacological effect. High oral bioavailability is often a key indicator to determine the drug-like property of bioactive molecules as therapeutic agents [51].

As for TCM, it has been believed that most compounds in the mixture fail to reach to the cellular targets since they lack appropriate pharmaceutical properties, especially the oral bioavailability [52]. Therefore, the valuation of oral bioavailability is indispensable to determine whether a compound is pharmacologically active in a TCM prescription. In CDF, 90 compounds were predicted satisfactorily to have high oral bioavailability (≥50%), which account for 28.1% of the total chemicals as shown in Table 1 and Table S1.

**Table 1:** The distribution of compounds with oral bioavailability in CDF formula.

Oral bioavailability	Number of compounds	Percentage (%)
≥90%	6	1.88
≥80%	15	4.69
≥70%	31	9.69
≥60%	53	16.56
≥50%	90	28.13

#### *Radix Salviae Miltiorrhizae*

As seen from Table 1, the compounds with high oral bioavailability (≥50%, 54/90) are mainly contained in *Radix Salviae Miltiorrhizae*, the “emperor” in the CDF formula. As the most abundant bioactive compounds [53], salvianic acid A, protocatechuic aldehyde and cryptotanshinone have the OB values of 78.2%, 53.4% and 57.4%, respectively. In addition, some other active compounds such as tanshinone IIB, isotanshinone IIA, IIB, and miltionone II all show a high oral bioavailability (≥50%) [54]. One exception is salvianolic acid B (Sal B), which has a very low oral bioavailability of 3.01%. Sal B, one of the most abundant constituents in Salvia species, shows good pharmacological effects on atherosclerosis [55], obstruction of regional cerebral blood flow [56] and platelet aggregation [57]. This raises the question that if this compound is not orally bioavailable, how it can exert desirable bioactivities in vivo. Further evidence shows that this compound is water soluble and can be rapidly metabolized *in vivo* to several products such as salvianic acid A (OB = 78.2%), isoferulic acid (OB = 67.7%) [58] and protocatechuic aldehyde (OB = 53.4%) [59], and quickly excreted into bile after oral administration [60]. All these data explains why Sal B does not have a high OB, and might also indicate that the pharmacological effects may not only be due to the Sal B itself but also its metabolites.

In addition to Sal B (OB = 3.01%), compounds tanshinone I (OB = 29.3%) and tanshinone IIA (OB = 20.3%) [61] are also considered as “Candidate Compounds” because these three molecules are the most abundant constituents in *Radix Salviae Miltiorrhizae* (∼>0.2%), although their OB values <50%.

#### *Panax Notoginseng*

29 compounds (2 overlap with *Radix Salviae Miltiorrhizae*) from *Panax Notoginseng* have a good oral bioavailability, including two documented bioactive molecules: dencichine (71.7%) and quercetin (51.0%), which have been reported to have good hemostatic [62], anti-cancer, and anti-thrombotic [63] effects. However, triterpene saponins notoginsenosides and ginsenosides, the main ingredients in *Panax Notoginseng*, are not orally bioavailable, as well as ginsenoside RF2 (OB = 36.4%) and other 18 saponins with OB <17.7% (ginsenoside Re). Considering that all these hydrophilic compounds have sugar groups and can be easily hydrolyzed into liposolubles, four main *in vivo* metabolites of these saponins, i.e., PPT (protopanaxatriol), PPD (protopanoxadiol), ginsenosides C-K and F1 [64], were additionally collected and their OB values are predicted to be 20.1%, 29.6%, 6.5% and 4.1%, respectively. The low OB for all the saponins explains why only 3.29% Rg1 and 0.64% Rb1 can be detected in rat serum for the orally administered ginsenosides [65], and why the intact ginsenosides, notoginsenosides and their metabolites are poorly absorbed in intestines or stomach [66], [67]. It is shown that the poor OB may be attributed to the following reasons: (1) pre-systemic elimination [68]; (2) gastrointestinal tract metabolism [69]; (3) potent efflux transport [70]; and (4) low membrane permeability. Among them, the membrane permeability might be a key factor in judging which a drug reaches the systemic circulation [69].

However, these findings are somehow contradictory to the existing data that the *Panax Notoginseng* exhibits incredible pharmacological activities, such as neuroprotection [71], antioxidation [72] and angiogenesis modulation [73]. Further analysis shows that, even at a very low dose, ginsenoside could exert strong pharmacodynamic effects [74], [75]. More interestingly, the ginsenoside metabolites by microflora, such as *Prevotella oris* [76], *Eubacterium A-44* [77], exhibited greater biological effects than their intact ginsenosides [78], [79].

This raises the question why *Panax Notoginseng* is effective while possessing ingredients have poor oral availability. To answer this, the compounds, including the intact ginsenosides and metabolites, i.e., compound K, PPD (major metabolites of PPDs), ginsenoside F1 and PPT (major metabolites of PPTs) [64] are also regarded as Candidate Compounds and their targets are further analyzed in Section 3.2.

#### *Borneolum*

9 compounds (3 overlap with *Radix Salviae Miltiorrhizae*) from *Borneolum* have good OB, including the most abundant compound *d-borneol* (OB = 81.8%). The *l-borneol* and *isoborneol*, both isomerides of *d-borneol*, also have good OB, 88.0% and 87.0%, respectively.

*Borneolum* is a widely used herbal in TCMs, which often acts as a ‘guiding herb’ – leading other drug(s) to the target tissues or organs [3]. The *Borneolum* is highly lipid-soluble, which can be absorbed rapidly in the gastrointestinal tract and penetrates the Blood Brain Barrier (BBB) [80]. Recent work found that it could increase the number and volume of pinocytosis vesicles in the BBB cells, thus accelerating the transport and leading medicine uplink [81], [82]. *Borneolum* can inhibit the function of P-gp, one of the most important efflux proteins in cell membrane [83]. As we know, the P-gp inhibition may have a profound effect on the pharmacokinetics of drug absorption [84], [85]. The molecular docking in this work shows that *d-borneol* has high binding affinity to human P-gp at the substrate recognition site [86] with a binding free energy of −6.34 kcal/mol. This might explain why the addition of *Borneolum* can promote the oral delivery of drug molecules [87] as well as the oral absorption of Radix Salviae Miltiorrhizae [88].

From the above, 101 compounds are finally regarded as “Candidate Compounds”, including 77 readily absorbed compounds, 17 intact ginsenosides, 4 main ginsenoside metabolites and 3 most abundant compounds.

### Target identification and validation

Cardiovascular disease has become a leading contributor to mortality in all over the world [89]. Currently, only a small number of proteins have been demonstrated as CVD targets for those approved drugs despite more than 230 proteins are confirmed related to the CVD [90]. The identification of novel targets for known drugs, as well as the discovery of cross-pharmacology relationships among targets has become urgent for the development of new target connections and novel drugs. Clearly, in a genome-wide way to search potential targets or target interactions, the “dry” experiment (computational method) should be the first choice since the “wet” experiment is time-consuming, expensive, and also limited in small scale [91].

In this work, a pharmacophore modeling technique was firstly applied to search potential targets based on the “Candidate Compounds”. In order to find as many as possible targets, the proteins whose fit score are ≥3.00 in the top 300 high-ranking proteins for each compound were considered as “Candidate Targets”. A total of 385 (Table S2) unique proteins were obtained as “Candidate Targets”, whereas 8 “Candidate Compounds” (compounds 216, 228, 254, 272, 274, 292, 299, 312) have no “Candidate Target” under this criterion. All these proteins were further subject to PharmGkb, DrugBank and TTD to check if they are related to CVD. Our results show that 42 positive targets and 4 “Candidate Compounds” (compounds 221, 255, 339, 344) do not have CVD-related targets.

In this pharmacophore-based target identification, the bioactivity was assessed merely by the atom and bond features of a tested molecule rather taking the whole ligand into consideration [92]. To improve the liability of the obtained models, the Candidate Targets related to CVD were further validated by molecular docking, and only those with binding free energy ≤−5.0 kcal/mol were kept as the “Potential Targets”. As a result, one Candidate Target (heme oxygenase 1) and 4 “Candidate Compounds” (compounds 209, 214, 273 and 338) are deleted, and some receptors are not targeted by certain compounds any more. For example, compounds 151 (salvianolic acid B) and 68 (tanshinol II) should not bind to ER-alpha (Estrogen receptor-alpha) since their binding free energies are 29.75 and 8.62 kcal/mol, respectively, although they are predicted to interact with this protein by the pharmacophore method. After this docking process, the number of interactions between the receptors (Candidate Targets related to CVD) and ligands (Candidate Compounds) is sharply reduced from 1580 to 735, with the “Potential Targets” and CVD-related “Candidate Compounds” to 41 (Table 2) and 85, respectively. The detailed interactions for these Candidate Compounds and Potential Targets with their ligands are shown in Table S2.

**Table 2:** The Potential Targets and the related diseases.

No.	Short Name	Gene Name	Protein Name	PDB	Related Diseases
1	ACE	ACE	Angiotensin-converting enzyme	1UZF	Coronary artery disease, Arteriosclerosis, Hypertension, Heart failure, Hypokinesia, Stroke, Thromboembolism
2	ACE2	ACE2	Angiotensin-converting enzyme 2	1R4L	Hypertension, Cardiovascular diseases
3	Aldose reductase	AKR1B1	Aldose reductase	2DUX	Cardiovascular diseases, Diabetes
4	Androgen receptor	AR	Androgen receptor	1GS4	Cardiovascular diseases
5	Ang	ANG	Angiogenin	1B1I	Cardiovascular diseases
6	CA2	CA2	Carbonic anhydrase 2	1I9P	Hypertension
7	Caspase-3	CASP3	Caspase-3	1RHR	Venous thrombosis
8	Cathepsin K	CTSK	Cathepsin K	1TU6	Atherosclerosis
9	Cathepsin S	CTSS	Cathepsin S	1NPZ	Atherosclerosis
10	Chymase	CMA1	Chymase	1T31	Hypertension, Coronary artery disease
11	CYP2C9	CYP2C9	Cytochrome P450 2C9	1R9O	Coronary artery disease, Heart diseases, Hypertension, Thromboembolism
12	eNOS	NOS3	Nitric oxide synthase, endothelial	3NOS	Angina pectoris, Thrombosis, Heart failure, Acute coronary syndrome, Cardiovascular diseases, Myocardial infarction, Hypertension
13	ER-α	ESR1	Estrogen receptor	1YIN	Hyperlipidemia, Coronary artery disease
14	ER-β	ESR2	Estrogen receptor beta	1NDE	Hyperlipidemia, Coronary artery disease
15	E-selectin	SELE	E-selectin	1G1T	Hypertension
16	F10	F10	Coagulation factor X	1MQ6	Coronary artery disease
17	F2	F2	Prothrombin	1TA2	Myocardial infarction, Thromboembolism
18	F7	F7	Coagulation factor VII	1DAN	Thromboembolism, Cardiovascular diseases
19	GR	NR3C1	Glucocorticoid receptor	1NHZ	Hypertension, Cardiovascular diseases
20	HMG-CoA reductase	HMGCR	3-hydroxy-3-methylglutaryl-coenzyme A reductase	3CD7	Myocardial infarction, Hyperlipidemias, Cardiovascular diseases, Arteriosclerosis, Hypertension
21	HSP90-α	HSP90AA1	Heat shock protein HSP 90-alpha	1UYH	Arteriosclerosis, Acute coronary syndrome
22	HSP90-β	HSP90AB1	Heat shock protein HSP 90-beta	1UYM	Arteriosclerosis, Acute coronary syndrome
23	iNOS	NOS2	Nitric oxide synthase, inducible	1NSI	Hypertension
24	LXR-α	NR1H3	Oxysterols receptor LXR-alpha	1UHL	Cardiovascular diseases, Hypertension, Coronary artery disease
25	LXR-β	NR1H2	Oxysterols receptor LXR-beta	1PQ6	Hypertension, Cardiovascular diseases
26	MIF	MIF	Macrophage migration inhibitory factor	1GCZ	Arteriosclerosis
27	MMP-9	MMP9	Matrix metalloproteinase-9	1GKD	Coronary artery disease, Heart failure
28	Mn-SOD	SOD2	Superoxide dismutase [Mn], mitochondrial	1XDC	Arteriosclerosis, Hyperlipidemia
29	MR	NR3C2	Mineralocorticoid receptor	2AA5	Hypertension, Hyperlipidemias
30	PDE4D	PDE4D	cAMP-specific 3,5-cyclic phosphodiesterase 4D	1Y2K	Heart failure, Arrhythmia
31	PPAR-α	PPARA	Peroxisome proliferator-activated receptor alpha	1K7L	Hypertension, Coronary artery disease, Hyperlipidemias, Cardiovascular diseases
32	PPAR-δ	PPARD	Peroxisome proliferator-activated receptor delta	1Y0S	Venous thrombosis, Hyperlipidemias
33	PPAR-ã	PPARG	Peroxisome proliferator-activated receptor gamma	1RDT	Hypertension, Cardiovascular diseases, Hyperlipidemias
34	RBP-4	RBP4	Retinol-binding protein 4	1RBP	Coronary artery disease, Arteriosclerosis, Hypertension, Hyperlipidemia
35	Renin	REN	Renin	2IKO	Coronary artery disease, Arteriosclerosis, Hypertension, Hyperlipidemia, Heart failure
36	RXR-α	RXRA	Retinoic acid receptor RXR-alpha	1FBY	Hypertension, Cardiovascular diseases
37	RXR-β	RXRB	Retinoic acid receptor RXR-beta	1H9U	Hypertension, Cardiovascular diseases
38	sPLA2-IIA	PLA2G2A	Phospholipase A2, membrane associated	1KQU	Myocardial infarction, Coronary artery disease
39	TGF-β1R	TGFBR1	TGF-beta receptor type-1	1RW8	Cardiovascular diseases, Hypertension
40	VDR	VDR	Vitamin D3 receptor	1DB1	Cardiovascular diseases, Hypertension
41	VEGFR-2	KDR	Vascular endothelial growth factor receptor 2	2OH4	Hypertension

After the pharmacophore modeling and docking validation process, 16 “Candidate Compounds” (compounds 216, 228, 254, 272, 274, 292, 299, 312, 221, 255, 339, 344, 209, 214, 273 and 338) are eliminated due to their low binding affinity with receptors. Further analysis shows that all the remained 85 CVD-related compounds are different from the above 16 CVD-unrelated chemicals: 1) The optimal pharmacophore models generally include at least 4 hydrophobic groups, 3 H-bond acceptors and 2 H-bond donors. However, most CVD-unrelated compounds have only 2 hydrophobic groups, 1 H-bond acceptor and 1 H-bond donor or less. 2) The docking simulations show that, in contrast to all the CVD-unrelated compounds (binding energies >−3.0 kcal/mol, average), the CVD-related compounds bind well to their targets (binding energies <−6 kcal/mol, average).

Compared with pharmacophore modeling, molecular docking might provide more reliable results. But these models are also suffered from an over estimation of the protein-ligand binding. Therefore, the binding of ligand with receptor in more realistic complex systems was further probed by molecular dynamics simulation and binding free energy analysis. Here, three systems, i.e., REN-15, REN-94, VDR-176 were collected based upon a careful consideration of following principles: 1) REN (renin) is an approved target for CVD drugs hydrochlorothiazide [93] and aliskiren [94], which is predicted to bind to compounds 15 (3α-hydroxytanshinone IIA) and 94 (dihydrotanshinone I) and adopted here as a positive control; 2) VDR (Vitamin D3 receptor) is an important predicted potential target, which binds to compound 176 (tanshinone IIA); 3) The three compounds are all key components in CDF with less than −5.0 kcal/mol docking binding energy.

MD trajectories of the three complexes in explicit solvent were calculated for 5 ns and the root mean square deviation (RMSD) of protein Cα backbone atoms reveals very small changes (∼1.8 Å), which is indicative of the satisfactory performance of the simulations. Subsequently, the absolute binding free energies of the three systems using the single-trajectory MM-PBSA method (Table 3) were calculated. In general, models with lower free energy are expected to be more stable than those with higher values. As seen from the Table 3, the low binding free energies (−21.24∼−27.14 kcal/mol) is indicative of high binding affinity of the three compounds to their targets. According to the energy individual components, the total electrostatic contribution (Δ*E*_ele_+Δ*G*_pb_) is unfavorable (positive) while the van der Waals and hydrophobic interaction contribution (ΔE_vdw_+ΔG_np_) is favorable (negative) for the binding. This is in agreement with the fact that the binding pocket of REN and VDR receptors are mainly composed of hydrophobic residues.

**Table 3:** Binding free energy estimates for each model.

Contribution	REN-94		REN-15		VDR-176	
	Mean	Std	Mean	Std	Mean	Std
**ΔE_ele_**	−7.65	3.86	−16.05	5.45	−5.48	1.77
**ΔE_vdw_**	−26.11	2.62	−32.50	2.45	−41.68	2.38
**ΔG_np_**	−4.17	0.24	−5.17	0.27	−5.53	0.09
**ΔG_pb_**	8.12	1.55	17.06	2.79	12.91	1.26
**ΔG_gas_**	−33.76	3.79	−48.54	5.71	−47.15	3.40
**ΔG_sol_**	3.95	1.49	11.89	2.71	7.38	1.28
**−TΔS**	15.88	7.35	13.91	7.06	18.53	5.91
**ΔG_bind_** a	−29.81	2.67	−36.65	3.75	−39.77	3.17
**ΔG_bind_** b	−27.14	−	−22.74	-	−21.24	-

Mean contributions are in kcal/mol.

a The predictions of binding energy do not include the entropy effect.

b The predictions of binding energy include the entropy effect.

### Network construction and analysis

Currently it has been recognized that CVD may be caused by a variety of complex reasons such as the disturbance of metabolism and genetic variations [89]. With the growing understanding of the complex disease, the focus of drug discovery has shifted from the well-accepted “one target, one drug” model designed toward a single target to a new “multi-target, multi-drug” model aimed at systemically modulating multiple targets in body [95]. Interestingly, as an empirical system of multicomponent therapeutics, TCM might have the potential of addressing a relationship between multicomponent and drug synergistic effects, which is capable of systematically controlling various diseases such as the angiogenic disorders [96], [97]. And the complex network analysis approaches might shed light on the mystery of TCM and uncover the synergistic effects among different components in the mixtures. And the application of network theory would be a very useful tool for us to visualize and analyze the interaction data to capture the complexity in a simple, compact, and illustrative manner. In the following part, we will discuss how the network pharmacology approaches have been applied in the TCM investigations.

#### C-cT and C-T networks: novel compound-target networks for CDF

**Figure 2:**
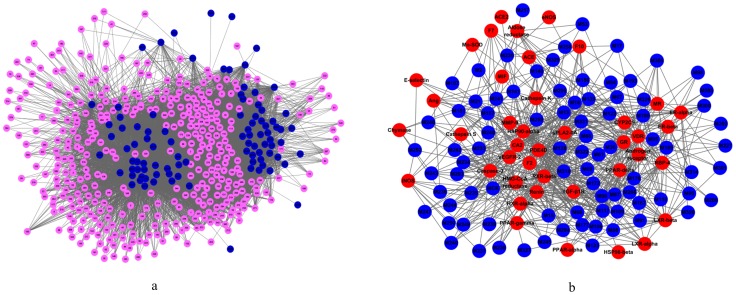
Compound-Target Networks. (a) C-cT Network. (b) C-T Network.

After deleting the 8 compounds with no targets, the resultant 93 “Candidate Compounds” and all their “Candidate Targets” were applied to generate a bipartite graph of Compound-candidate Targets interactions, in which a compound and a target are connected to each other if the protein is a Candidate Target of the Candidate Compound, giving rise to a “C-cT Network”. Figure 2a shows a global view of C-cT Network with color-coded nodes: Candidate Compounds (blue), Candidate Targets (pink).

The C-cT Network consists of 478 nodes and 9220 edges, with 93 Candidate Compounds and 385 Candidate Targets. The 93 compounds display a total of 9220 interactions with their targets. Most Candidate Compounds target only a few Candidate Targets, but some have many Candidate Targets. Compound 235 (ginsenoside Rb1) exhibits the highest number of Candidate Target interactions (180), followed by compound 236 (ginsenoside Rb2) with 179 Candidate Targets, and compound 247 (ginsenoside Ro) with 178 Candidate Targets. Compounds 209 (1-methyl-5-isopropenyl cyclohexene1), 221 (3-ethyl-2, 4-pentylene alcohol), 255 (butyl cyclobutane) and 344 (β-terpineol) all have the least number of Candidate Targets (only 1). This indicates that the C-cT Network that encodes a compound-target space is biased toward certain drug compounds. Likewise, the Candidate Targets also display rich landscape of interacting compounds (9220 in total, mean value: 23.9). Among the 385 Candidate Targets, Proteins 168 (glutathione S-transferase P) and 185 (heat shock protein 90 kDa alpha) possess the largest number of interacting Candidate Compounds (81). The following order is Proteins 67 (carbonic anhydrase 2) and 374 (tyrosine-protein phosphatase non-receptor type 1) with 80 and 79 connected Candidate Compounds, respectively. Higher-degree Candidate Compounds and Candidate Targets in the C-cT Network are preferentially connected to each other rather than being distributed homogeneously throughout the network, leading to a much smaller giant component size than expected. Therefore, the C-cT Network represents an intermediate structure between a completely random network with a very large giant component and a functionally fully segregated network broken into isolated clusters. Clearly, the C-cT Network might be a useful compendium to reflect the Candidate Compounds and Candidate Targets in the treatment of CVD. The general network properties of the C-cT Network are listed in Table 4.

**Table 4:** The general network properties of the C-cT and C-T Network.

Network	Number of nodes	Number of edges	Avg. degree	Network density	Network centraliazation	Characteristic path length	Shortest paths	Network heterogeneity
**C-cT**	478	9220	38.577	0.081	0.298	2.429	228006	1.110
**C-T**	126	735	11.667	0.093	0.320	2.485	15750	0.831

Furthermore, extracted from the bipartite C-cT Network graph, we generated a C-T Network (Compound-Potential Target Network, Figure 2b) projection by connecting the 85 validated CVD-related Candidate Compounds (after deleting those 4 compounds with binding free energy ≤−5.0 kcal/mol with their receptors) and their 41 Potential Targets (Table 2, Figure 2b). Table 4 lists the general network properties of the C-T Network, which is composed of 126 nodes and 735 edges, with 85 Candidate Compounds and 41 Potential Targets. Figure 2b shows the topology of the C-T Network with colour-coded nodes: Candidate Compounds (blue), Potential Targets (red). The average number of Potential Targets per Candidate Compound is 8.6. Of all the 85 Candidate Compounds, 31 have a relatively strong interaction with ≥10 Potential Targets, and 11 compounds bind to more than 15 Potential Targets. Compound 13 (neryl formate) exhibits the highest number of interactions with 25 Potential Targets, following on are compounds 331 (neryl acetate, 21 targets), 117 (manool, 21 targets), 128 (neocryptotanshinone, 20 targets) and 69 (tanshinol I, 20 targets). This also indicates that C-T Network is biased toward specific drug compounds.

Similarly as the whole C-cT Network, many Potential Targets are targeted by more than one Candidate Compound. HSP90-alpha (heat shock protein 90 kDa alpha), PDE4D (cAMP-specific 3,5-cyclic phosphodiesterase 4D), VDR (vitamin D receptor) and RXR-beta (retinoid×receptor, beta) are examples of highly connected Potential Targets, whose numbers of Candidate Compounds are 51, 44, 41 and 40, respectively. But the ACE2 (angiotensin-converting enzyme 2) has only one interactional Candidate Compound. The average number of Candidate Compounds per Potential Target is 17.9, indicating that many proteins that are related to CVD might share similar binding patterns with the ligands. Interestingly, the C-T Network exhibits similar features of drug-target interactions as those of C-cT Network, which might further demonstrates the reasonability of the obtained network.

#### C-T network: pinpointing the key players of CDF for CVD from the fundamental global and local properties

Network data structures are amenable to many sophisticated forms of computational analysis which can uncover important, nonobvious properties of nodes and the relationships between them [98], [99]. The topological analysis of the networks may offer insights into the biologically relevant connectivity patterns, and pinpoint highly influential compounds or targets.

A general overview of the global topological properties of the C-T Network comes from the diameter and the average distance of the network. A diameter of 5.0 with an average distance of 2.48 suggests a highly connected network, in which Candidate Compounds and Potential Targets are strongly functionally interconnected. To support this suggestion, we also analyzed the centroid and eccentricity (Table S4). The centroids of 61 nodes are larger than the network average centroid (−66.7), and 100 nodes have the eccentricity value larger than the average value of 4.12. These results demonstrate that our C-T Network is a highly linked network.

Furthermore, the node degree (the number of connections or edges the node has to other nodes), as one of the most basic quantitative properties of a network, is also investigated. The highly connected nodes are referred to as hubs [100]. The node degrees of C-T Network follow an interesting distribution, i.e., most nodes display a medial number of interactions; others are highly or loosely connected. This indicates that C-T Network interactions are not generated at random and they may encode clinically relevant associations. Of all the 85 Candidate Compounds, 31 compounds possess degree larger than 10 under an average value of 8.6 in which 16 are known active compounds. Whereas, in the top 43 (half of the 85 total) compounds, 24 are known active ones in the C-T Network (as shown in Table 5). These Candidate Compounds participating in more interactions than other components are the hubs in this C-T Network.

**Table 5:** The betweeness and node degree of Candidate Compounds.

Compounds	Betweenness	Degree	Compounds	Betweenness	Degree
M2	1.9130	3	M206	0.7509	2
***M13***	***724.1483***	***25***	M217	0.0000	1
***M15*** *	***180.7998***	***13***	M227	4.6731	4
M17	21.6577	5	**M230** *	**455.5151**	**9**
***M18***	***112.4945***	***11***	***M233*** *	***116.7896***	***13***
***M21***	***359.3636***	***14***	**M234** *	**61.1274**	**10**
***M36***	***111.0346***	***12***	**M235** *	**93.3910**	**9**
M53	16.4952	4	M236	24.0610	5
M61	16.2719	5	M237	26.0983	6
**M66**	**68.6507**	**10**	M238	9.0711	5
M67	19.2309	5	M239	15.1011	5
***M68*** *	***291.2255***	***19***	M240	24.9245	6
***M69*** *	***350.7039***	***20***	**M241** *	**92.3841**	**8**
**M80** *	**60.3365**	**9**	***M242*** *	***104.5600***	***10***
**M87**	**69.4337**	**10**	M243	67.0867	7
M88	43.5437	7	M244	52.0521	7
***M91***	***123.2400***	***13***	M245	16.8538	6
M94	14.2769	4	M246	45.1483	5
M95	0.0000	1	M247	2.3093	3
M98	7.4776	4	M248	0.0000	1
***M99***	***147.0782***	***14***	M249	26.8981	6
***M106*** *	***230.6302***	***15***	M250	37.1891	7
M107	0.6961	2	M252	17.6270	3
***M109*** *	***244.4809***	***18***	M258	100.4710	5
**M110** *	**61.3330**	**10**	***M259***	***90.7898***	***11***
***M117***	***518.7937***	***21***	**M286**	**62.3123**	**9**
M122	13.3008	4	M293	45.8579	9
**M123** *	**51.7626**	**8**	***M294*** *	***138.5526***	***13***
***M128***	***448.0906***	***20***	**M295** *	**67.9490**	**9**
***M139***	***342.8197***	***18***	M300	47.3511	9
***M141*** *	***242.6144***	***13***	M301	10.6174	4
***M148*** *	***169.9105***	***12***	M302	5.9018	4
***M151*** *	***390.0531***	***16***	M305	23.6586	7
***M156***	***116.6271***	***11***	M314	44.8343	8
***M157***	***198.5621***	***13***	**M316**	**86.7339**	**9**
M167	10.6765	5	M320	7.3409	3
M172	31.7887	7	M322	12.8785	4
**M176** *	**48.0524**	**8**	M326	2.1035	2
***M177*** *	***293.8129***	***16***	M327	1.7637	2
**M180** *	**80.7252**	**10**	M329	28.0748	6
M189	18.8204	5	***M331***	***437.6101***	***22***
M193	35.0662	6	***MCK*** *	***144.5436***	***13***
M205	1.0506	2			

***Bold and italic figure***: compounds with both high degree and betweenness value in the top 30 compounds.

**Bold figure:** compounds with both high degree and betweenness value in the top 43 (half of the 85 total) compounds.

* **:** compounds which have been demonstrated actively in the CDF formula.

Another fundamental property of network nodes is the “betweenness”, the capacity to be located in the shortest communication paths between different pairs of nodes in the network [100]. This property is also defined as traffic. High traffic nodes are referred to as network bottlenecks. Some previous work including ours has demonstrated the potential biological relevance of high traffic nodes with regard to their functional coordinating roles and phenotypic effects [101], [102]. In the C-T Network, of all 85 Candidate Compounds, 16 are known active compounds in the top 30 compounds that have higher betweenness, and 25 in the top 43 (half of all), as shown in Table 5.

**Figure 3:**
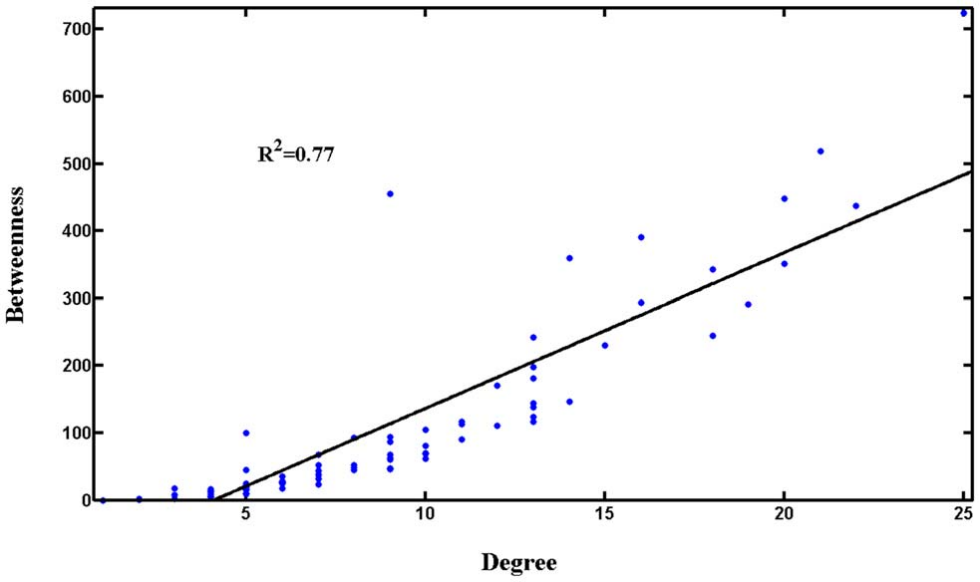
Relationship between node betweenness and degree distribution.

Generally, nodes (Candidate Compounds in the present network) which have both higher degree and betweenness would be more important [103]. Interestingly, we observe that the betweenness and degree values in the C-T Network are strongly correlated (Figure 3), with a correlation coefficient of R^2^ = 0.77. Those nodes (Candidate Compounds) which have higher degree would have larger betweenness, and 40 of the top 43 Candidate Compounds have both high degree (≥8) and betweenness (≥48.05); and the number is 18 of the top 20 compounds. This means that C-T Network hubs tend to encode the bottlenecks, and influence different network regions through both direct and indirect interactions.

Surely, Candidate Compounds with higher degree and betweenness would be key players in the CDF. 10 among the 18 key Candidate Compounds with high degree and betweenness (Table 5) have been well demonstrated active in the CDF formula; and the top 40 key Candidate Compounds with high degree and betweenness include 23 reported active compounds. For instance, compound 148 (salvianic acid A) can dilate the isolated coronary artery, increase the coronary flow rate and expand the blood vessel [18]; compounds 177 (tanshinone IIB) [19] and 230 (dencichine) [20] are effective to inhibit the platelet aggregation. This also indicates that the network-based analysis is capable of extracting the key components in this herbal medicine. In addition, it is worth noting that some compounds, whose activities are still unknown, should be a key area of consideration in the future study of CDF. Especially, four Candidate Compounds, i.e., compounds 117 (manool), 128 (neocryptotanshinone), 157 (salvianolic acid J), and 99 (epidanshen spiroketal lactone), might be novel leads for treatment of CVD and worth further research. Interestingly, it is found that 28 of the 40 key ingredients are contained in *Radix Salvia Miltiorrhiza*, which further demonstrates that this emperor drug plays a key role in the CDF formula.

Besides, evidence suggests that some chemicals can exert pharmacological activities by acting on gastrointestine [104], thus, the intestinal metabolites of ginsenosides are also incorporated to understand the mechanism of *Panax Notoginseng*. Ginsenosides Rd, C-K, F2 and PPD are metabolites of ginsenoside Rb1, while Rh2 and PPD are those of Rg3; for Re, its metabolites are Rh1, Rg2, F1 and PPT [64]. Network analysis shows that the metabolites ginsenoside C-K and PPD play more important roles than the original Rb1. Also, PPD is more important than Rg3, and F1, Rg2 and PPT are more important than Re. All these may suggest that the metabolites, not the original ginsenosides, exert main pharmacological activities in the treatment of CVD.

The network analysis also shows that, as two major intestinal metabolites of PPDs [64], C-K and PPD play more important roles than all the original PPDs. And F1 (the major intestinal metabolite of PPTs) [64] is more important than PPTs. All these indicate that the intestinal ginsenoside metabolites might be the main active ingredients of *Panax Notoginseng* in the treatment of CVD.

Among the ten most abundant compounds in CDF (tanshinone IIA, Sal B, cryptotanshinone, protocaterchuic aldehyde, salvianic acid A, tanshinone I, notoginsenoside R1, ginsenoside Rg1, ginsenoside Rb1 and d-borneol) [3], three compounds, i.e., Sal B, protocaterchuic aldehyde and salvianic acid A, are included in the top 20 key players (Candidate Compounds), which implies that these 3 compounds may play more important roles than other ones in treating the disease. In the top 20 players, CK and PPD reflect greater contribution of Rb1, and tanshinone IIA and cryptotanshinone also show greater contribution than others. This implies that the six compounds of Sal B, protocaterchuic aldehyde, salvianic acid A, ginsenoside Rb1, tanshinone IIA and cryptotanshinone are probably the most important components for the treatment of CVD in a real patient.

In addition, we also analyzed the Potential Targets of these Candidate Compounds (with detailed information shown in Table S3). Of the top 20 Potential Targets (41 in total) with high degree or betweenness, 18 have both high degree and betweenness. Thus, they could be key targets for CDF, and play key roles in CVD therapy. Amazingly, *Radix Salvia Miltiorrhiza* and *Panax notoginseng* both target all these 18 proteins, which also explains the principal or adjuvant functions of these two herbs in CDF. All these data indicate that the CDF treats the cardiovascular disease based on the synergistic interactions of different components.

#### C-T network: illustrating the mechanisms of the CDF on CVD based on the compound-target interactions

Advances in pathophysiological research suggest that the CVD continuum begins with risk factors that initiate the process that leads to tissue damage. The pathophysiological continuum includes the oxidative stress, endothelial dysfunction, inflammatory processes, vascular remodeling in the initiation and continuation of CVD, thrombosis process, dyslipidemia and dysarteriotony, etc [105]. Collectively, these risk factors might alter the expression of proteins in multiple cellular pathways, leading to changes at the individual cell level, the tissue level and, ultimately, the disease state. The strategy behind the modern pharmaceuticals is to restore the healthy state by inhibiting a molecular target that is central to the disease mechanism. However, a greater understanding of the CVD network reveals that inhibition of an individual target is insufficient to restore the system to the healthy state. In these cases, modulating the activity of multiple targets would be unquestionably required to achieve optimal therapeutic benefit [106]. The action mechanism for a TCM is most probably due to that the active compounds target at multiple proteins in the biological network and then the biological system would attain new equilibrium in order to reduce the harmful impact. It is most likely that these targets make up a great and interlinked network so that these building blocks could function as a whole.

In our study, there are 41 validated Potential Targets which have been annotated to have significant relationship with the pathological process of CVD. All these proteins might mediate at every stage along the CVD continuum. For example, F2 (thrombin), F7 (coagulation factor VII), F10 (coagulation Factor Xa) and PPAR-delta (peroxisome proliferator activated receptor delta) play important roles in the thrombosis process; while LXRs (liver×receptor alpha and beta), HMG-CoA reductase (3-hydroxy-3-methylglutaryl-coenzyme A reductase), PPAR alpha, delta, gamma, ERs (estrogen receptor alpha and beta), RBP4 (retinol-binding protein 4), TGF-β1R (TGF-beta receptor type-1) and Mn-SOD (superoxide dismutase [Mn], mitochondrial) are closely concerned with the lipid metabolism and peroxidation, and may cause dyslipidemia. Interestingly, 39 of all these validated targets (except eNOS and ACE2) are for *Radix Salvia Miltiorrhiza*, the emperor of CDF. Clearly, the compounds interacting with these receptors associated with thrombosis and hyperlipidemia could lead to inhibition of the blood coagulation, activation of the fibrinolysis, inhibition of the platelet aggregation and tackiness, decrease of the plasma viscosity, and ultimately cure of the thrombosis.

Proteins eNOS (nitric oxide synthase, endothelial), CYP2C9 (cytochrome P450, family 2, subfamily C, polypeptide 9), HSP90s (heat shock 90 kDa protein 1, alpha and beta), PPAR alpha, gamma and MIF (macrophage migration inhibitory factor) are all well related to the vasodilatation, reactive oxygen species (ROS) and inflammation, the control of which will lead to the improvement of endothelial and vasomotor dysfunction, inhibition of inflammatory process and prevention damage of the inflammatory factor to the blood vessel and cardiac muscle. And PPAR alpha, gamma, E-selectin, GR (glucocorticoid receptor), LXR-beta, alpha, RXR-alpha (retinoid×receptor alpha) and AR (androgen receptor) are all concerned with hypertension. So through the modulation to these proteins, the CDF may achieve the antihypertensive curative effect.

The proteins concerned with vasoconstriction are rennin, ACE and chymase, VDR (vitamin D receptor) and VEGFR-2 (vascular endothelial growth factor receptor 2), and the regulation of them may cause hemangiectasis, and then lower blood pressure. Caspase-3, MMP-9 (matrix metalloproteinase 9), MR (mineralocorticoid receptor), TGF-β1R, Ang (angiogenin), AR (aldose reductase), PDE4D (cAMP-specific 3, 5-cyclic phosphodiesterase 4D) and sPLA2-IIA (phospholipase A2 membrane associated) are somewhat related to proliferation and apoptosis of vascular smooth muscle cells. Proliferation of intimal vascular smooth muscle cells is an important component in the development of atherosclerosis; therefore the regulation of these proteins may inhibit the proliferation of vascular smooth muscle cells and further control the process of CVD. AR (aldose reductase), MMP-9, Cathepsin K and S are involved in vascular remodeling, thus by regulating of these targets may achieve the goal of reducing vascular remodeling and cure atherosclerosis and hypertension.

For *Panax Notoginseng*, there are 36 Potential Targets, 34 of which are overlapped with those of *Radix Salvia Miltiorrhiza*. Interestingly, the 21 compounds from *Panax Notoginseng* participate in all the six pharmacological processes as previously mentioned. This indicates that *Panax Notoginseng* also has anticoagulant, antihyperlipidemia, antihypertensive, anti-inflammatory effects and inhibits the vascular remodeling and proliferation of vascular smooth muscle cells. Notably, there are three unique potential targets of its own, i.e., ACE2 and eNOS. The ACE2 can catalyse the conversion of angiotensin I to the nonapeptide angiotensins (1–9), or the conversion of angiotensin II to angiotensin, so the regulation of ACE2 may have an effect on hypertension or ischemic cardiovascular disease. While the eNOS has a central role in the regulation of vascular smooth muscle tone. Its modulation may also lead to the proliferation of vascular smooth muscle. Therefore, it can be deduced that the action mechanism of this medical composition is that the CDF systematically controls the CVD via potentially synergistic interactions of the active compounds [107], where *Radix Salvia Miltiorrhiza* is the key while *Panax Notoginseng* adjuvant. In addition, the result shows that the targets of *Radix Salvia Miltiorrhiza* focus on the whole cardiovascular system, while to the *Panax Notoginseng*, except the enhancement, it places emphasis on the modulation of vascular smooth muscle cells. These data also explain why in the CDF, *Radix Salvia Miltiorrhiza* is used as the emperor, while *Panax notoginseng* is used as the minister drug to enhance the pharmacological actions of *Radix Salviae Miltiorrhizae*, which also approves the reasonability of the CTM theory in construction of a formula.

#### C-P (compound-pathway) network: compounds in the C-T network impact diverse clinically-relevant signal pathways

Different network regions may underlie different biological pathways, processes or cellular localizations. Drug action is not only related to its targets, but also affects various metabolic enzymes, transporter proteins, as well as the downstream effects of drug action and pathways related to the specific disease. Multiple compounds can jointly perturb the same disease-related signal pathways [108]. A more detailed characterization of these relationships may offer a viable strategy to explain the side effects, to improve the treatment effectiveness and to explore the potential drug repositioning. For this reason, to understand the therapeutic mechanisms of a drug, it is also critical to identify the signal pathways its targets participate in.

**Figure 4:**
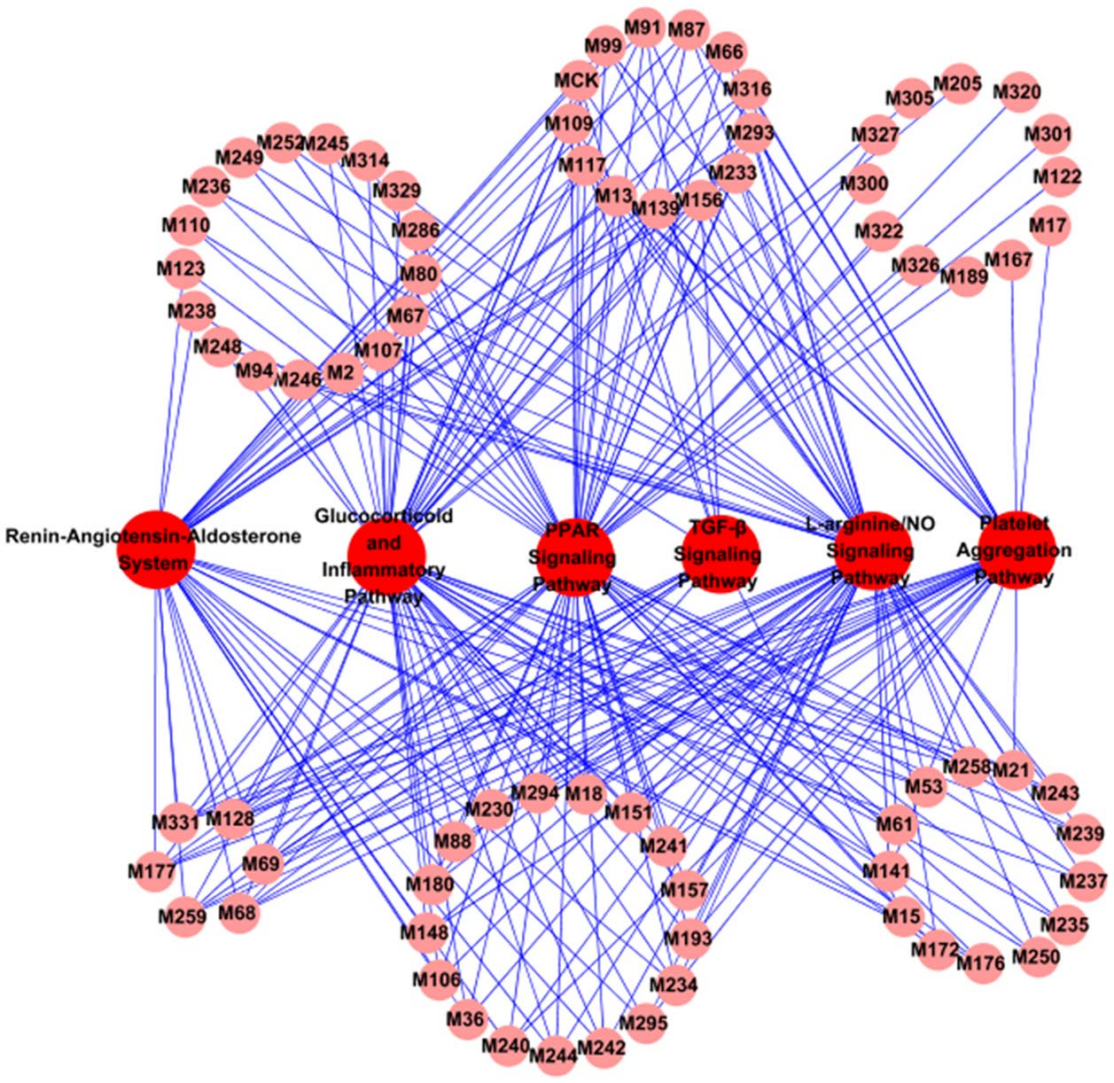
C-P Network.

To understand the therapeutic mechanisms of CDF, we have extracted the most highly related pathways associated with CVD in KEGG (www.genome.jp/kegg), resulting in six pathways, i.e., Renin-Angiotensin-Aldosterone System, Glucocorticoid and Inflammatory Pathway, PPAR Signaling Pathway, Platelet Aggregation Pathway, L-arginine/NO Signaling Pathway and TGF-β Signaling Pathway. The Renin-Angiotensin-Aldosterone System plays an important role in regulating the blood volume and systemic vascular resistance, which together influence cardiac output and arterial pressure [109]. The Glucocorticoid and Inflammatory Pathway participates in the regulation of inflammation [110], while the PPAR Signaling Pathway plays an important role in the clearance of circulating or cellular lipids via the regulation of the gene expression involved in lipid metabolism [111]. The Platelet Aggregation Pathway is related to the platelet activation and coagulation [112], and the L-arginine/NO Signaling Pathway is concerned with the nitric oxide biosynthesis and modulates the vascular endothelial function [113]. As to the TGF-β Signaling Pathway, it exerts pleiotropic effects on cardiovascular cells, regulating cell growth, fibrosis and inflammation, and participating in the pathogenesis of hypertension, atherosclerosis, cardiac hypertrophy and heart failure [114]. Subsequently, we mapped the Candidate Compounds onto these six KEGG pathways and generated a bipartite graph of C-P Network (Compound-Pathway Network, Figure 4), in which a Candidate Compound and a signal pathway were linked if the compound targets the proteins appeared in the signal pathways.

After discarding the 7 compounds not participating in any signal pathway, the C-P Network consists of 84 nodes and 254 edges, with 78 Candidate Compounds and 6 KEGG pathways. Figure 4 shows the global view of the C-P Network with color-coded nodes: Candidate Compounds (pink), signal pathways (red). As can be seen clearly, all the Candidate Compounds are found being involved in the related pathways.

It is interesting to find that 60 Candidate Compounds included in the C-P Network are linked to the PPAR Signaling Pathway. And Glucocorticoid and Inflammatory Pathway is shown as the second most targeted pathway interacting with 58 Candidate Compounds, with L-arginine/NO Signaling Pathway as the third one interacting with 57 Candidate Compounds. And 35 Candidate Compounds are found to perturb the Renin-Angiotensin-Aldosterone System. As to the Platelet Aggregation Pathway and TGF-β Signaling Pathway, their numbers of interacted Candidate Compounds are 31 and 13, respectively. Figure 4 displays a detailed view of relationships between multiple drugs and specific pathways. Actually, CDF has been reported to influence these pathway-related physiological processes [5], [6], [115]. For example, Zhou et al. have reported that the inhibition of tanshinone IIA to platelets and coagulation activity might lead to the regulation of the Platelet Aggregation Pathway and then improve the inflammation damage of vesselsin immune vasculitis [115].

From this, we speculate that CDF probably modulates the PPAR Signaling Pathway, Glucocorticoid and Inflammatory Pathway and L-arginine/NO Signaling Pathway, and in this way exhibits its pharmacological effects, and the cardiovascular effective roles primarily include anti-hyperlipidemia, antiinflammation and the improvement of endothelial and vasomotor functions. The regulation of the Renin-Angiotensin-Aldosterone System may reduce the arterial pressure, ventricular afterload and blood volume, as well as inhibit and reverse the cardiac and vascular hypertrophy. The modulation of the Platelet Aggregation Pathway may inhibit the activation of platelet and prevent the formation of thrombus. The reconciliation to the TGF-β Signaling Pathway may contribute to fibrosis in hypertension and cardiac damage.

The six pathways were interdependent with each other through the Candidate Compounds, which further indicates that CDF can exert synergistic influences on different pathways. In addition, a Candidate Compound may target different proteins involved in the same pathway or different pathways, which also illustrates the mechanism of multiple targets for a TCM. Since the six pathways are closely associated with inflammation, blood coagulation, vasodilatation, blood pressure, fibrillation, and blood lipid, we speculate that the CDF formula probably perturbs the pathways, and thereby displays the anti-inflammatory, anticoagulant, vasodilator, antihypertensive, anti-fibrillation and anti-hyperlipidemia activities [6], [7].

#### T-D (target-disease) network: various therapeutic effects and new indications of CDF

The tremendous medical value in finding new indications for existing drugs has been well recognized by the industry for many years. Exploring the potential therapeutic effect of certain drugs may be a very efficient strategy in drug development. Thus, we also need to know which targets of a drug are relevant to the new therapeutic effects and its likelihood of becoming effective drug in the new positions. It is known that most complex diseases are not caused by changes in a single causal gene but by an unbalanced regulating network resulting from the dysfunctions of multiple genes or their products. Different complex diseases associated with the same disease gene or gene products more often tend to share a protein-protein interaction (PPI) and biological process. So when a drug acts on the proteins associated with different diseases, it may show different therapeutic effects.

**Figure 5:**
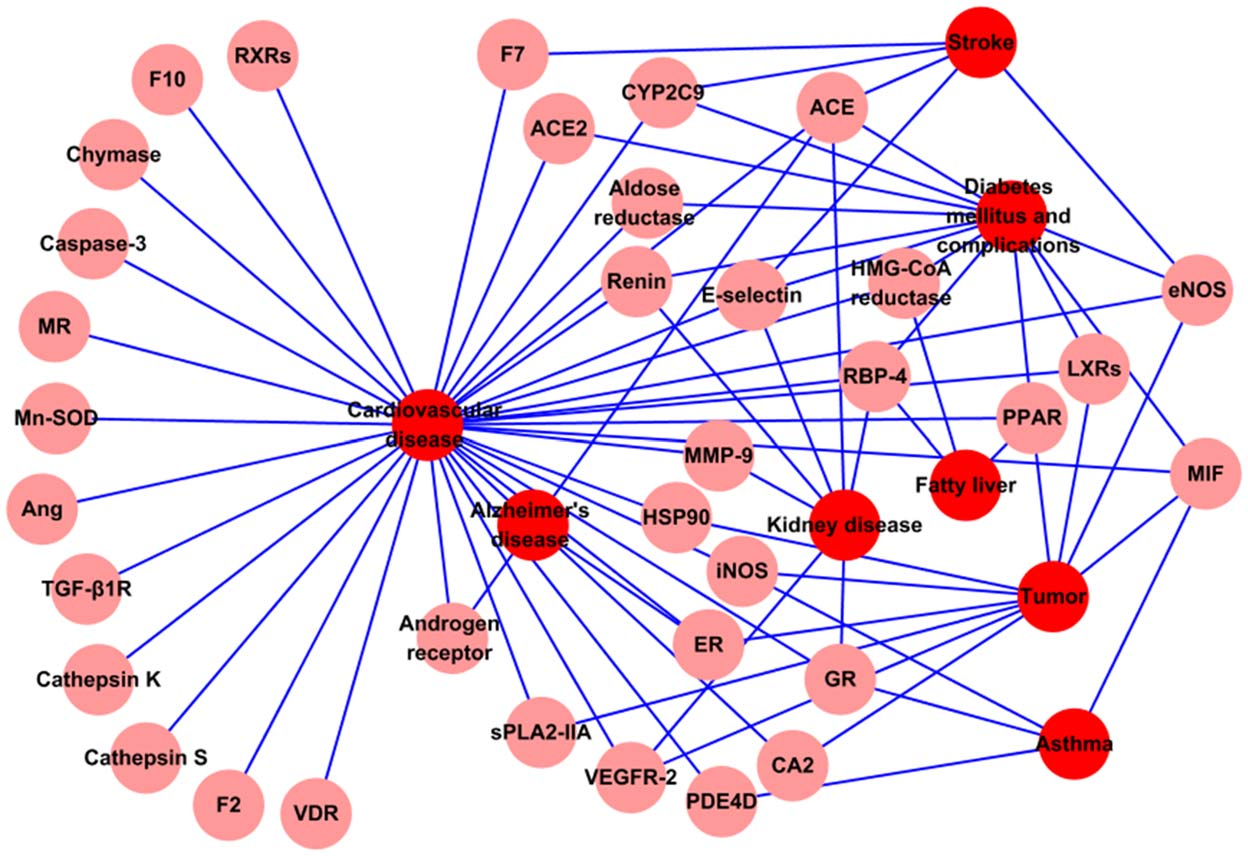
T-D Network.

Therefore, we have constructed the T-D Network (Figure 5) to find novel therapeutical effects of CDF, based on the assumption that the certain drug acting on same protein associated with different diseases in a network may cause different diseases. In the Target-Disease Network, the disease is linked to the Potential Target if the target has relationships with the disease, with color-coded nodes: disease (blue), Potential Target (pink).

Metabolomic data may provide useful information to explore the underlying mechanisms of the diseases, especially for the metabolic diseases caused by disorders of metabolism [116]. In this work, among all the 41 Potential Targets, some proteins have been confirmed to be closely related to the metabolism. For example, the aldose reductase, LXRs, PPARs, HMG-CoA reductase and RBP-4 can regulate glucose and lipid metabolisms. Indeed, the genesis and development of diabetes mellitus, hypertension, hyperlipidemia, fatty liver and tumor are all involved in the disorders of glucose, lipid and energy metabolisms. Therefore, the CDF may have potential pharmacological effects on the metabolic diseases since they can act on the metabolism-related targets.

Moreover, these 41 Potential Targets also participate in the occurrence and progress of some other diseases. Here, collecting all diseases related to the 41 Potential Targets from TTD [27], we summarized as follows: ACE, ACE2, aldose reductase, CYP2C9, eNOS, E-selectin, HMG-CoA reductase, LXRs, MIF, PPARs, RBP-4 and rennin are involved in diabetes mellitus and the complications, while the ACE, E-selectin, GR, MMP-9, RBP-4, rennin and VEGFR-2 are for the kidney disease; The ACE, CYP2C9, eNOS, E-selectin, and F7 play roles in stroke, while the ACE, ER and AR (androgen receptor) are associated with Alzheimer's disease. Meanwhile, the occurrence of tumor is concerned with CA2, eNOS, ER, HSP90, iNOS, LXRs, PPARs, MIF, sPLA2-IIA and VEGFR-2, and the function of GR, iNOS, MIF and PDE4D are involved in asthma; And also, the HMG-CoA reductase, PPARs and RBP-4 affect the development of hyperlipidemia fatty liver. All these findings suggest that CDF might regulate the whole body system by a complex protein-protein interaction network, thus affecting different diseases.

In fact, this hypothesis has been demonstrated in some recent work. For example, *Radix Salvia Miltiorrhiza* has been used as a standard treatment in stroke in China [117]. The CDF can also improve the vascular state in diabetic patients by reducing the activity of platelet membrane glycoproteins in a study of 82 patients [118]. *Radix Salvia Miltiorrhiza* is helpful for recovery of renal function in the early stage of renal transplantation [119] and plays an important role in the treatment of primary nephrotic syndrome in children [120]. In the treatment of bronchial asthma, the CDF is comparable to ketotifen (an allergy drug) without significant side effects [121]. In addition, in the way of delaying brain aging, improving cognitive and memory functions and preventing the Alzheimer's Disease, CDF also has significant curative effect [122]. Our work, from a molecular systems level, explains why the CDF is also effective in treatment of various diseases except for CVD. Clearly, these interesting associations provide a new clue for the Chinese traditional herbs study, and complement the corresponding experimental studies.

## Conclusion

Traditional Chinese Medicine is a unique (independent) system of theory, diagnosis and treatment tools in terms of composition or from the pharmacodynamics. In TCM, as a chief means of treating diseases clinically, TCM prescriptions usually consist of several medicinal herbs called “Fufang” in Chinese, based on the principle of “Jun-Chen-Zuo-Shi”-: “Jun” (emperor) treats the main cause or primary symptoms of the disease; “Chen” (minister) enhances the actions of “Jun” or treats the accompanying symptoms; “Zuo” (adjuvant) not only reduces or eliminates the possible toxic effects of the Jun or Chen, but also treats the accompanying symptoms; “Shi” (courier) helps to deliver or guide the other herbs to the target organs [47].

Compared with western medicine, the TCM approach treats the function and dysfunction of living organisms in a more holistic way. However, the complexity of the chemical components and the actions *in vivo* would lead to great difficulties to elucidate the molecular mechanisms of TCM. How to understand the TCM system as a whole (that is, the external signs) and the internal changes in the relevance has, thus, become the “bottleneck” of modern TCM study.

In this work, we proposed for the first time a new modeling system, combining oral bioavailability screening, multiple drug targets prediction and validation, network pharmacology to probe the efficiency of a representative TCM recipe Compound Danshen Formula for the treatment of CVD. Our results suggest that *Radix Salviae Miltiorrhizae* is the emperor in this formula, whereas *Panax Notoginseng* and *Borneolum* could serve as minister and courier drugs, which not only makes a better understanding of the mechanisms of CDF, but also provides modern insight for interpreting the theory of “jun-chen-zuo-shi” of TCM. Our main findings are:

1. A novel strategy is constructed to investigate into the mechanisms of action of the CDF from chemical, genomic and pharmacological data in an integrated framework.
2. The system can pinpoint out the key players of this formula, its active components and the corresponding targets, based on the synergistic interactions of the compounds, targets and pathways, which will be helpful for therapeutic applications of Chinese traditional herbs.
3. The developed system can be effectively applied to interpret the essence of Chinese medicine “synergy” and the most influential theory of “jun-chen-zuo-shi” of TCM. It provides a new way to hold the key to the inter-relationship between complex diseases and drug interventions through the network target paradigm for TCM.

Despite these potentially interesting associations, cautious interpretation is warranted as these findings relied on statistical analysis. Moreover, experimental testing of these hypotheses will be required to support further assessments of potential clinical application.

## Supporting Information

Table S1 **The oral bioavailability of all compounds.**
(XLS)

Table S2 **The Candidate Compounds and their Candidate Targets and Potential Targets.**
(XLS)

Table S3 **The betweenness and degree value of the Potential Targets.**
(XLS)

Table S4 **The CentiScape Centroid, CentiScape Eccentricity and Eccentricity of the Candidate Compounds and their Potential Targets.**
(XLS)
